# Antimüllerian Hormone as a Tool to Predict the Age at Menopause

**DOI:** 10.3390/geriatrics8030057

**Published:** 2023-05-19

**Authors:** Efstathia Chatziandreou, Andreas Eustathiou, Areti Augoulea, Eleni Armeni, Nikoletta Mili, Ioannis Boutas, Nikolaos Tsoltos, Antigoni Kapetanaki, Sofia Kalantaridou

**Affiliations:** 1 2nd Department of Obstetrics and Gynecology, National and Kapodistrian University of Athens, GR-11528 Athens, Greece; 2 3rd Department of Obstetrics and Gynecology, National and Kapodistrian University of Athens, GR-11528 Athens, Greece; 3 Hormonal Laboratory, Aretaieio Hospital, National and Kapodistrian University of Athens, GR-11528 Athens, Greece

**Keywords:** anti-Müllerian hormone, climacteric symptoms, menopausal symptoms, menopause

## Abstract

This study aimed to assess an eligible cut-off value of anti-Müllerian hormone (AMH) to detect ovarian senescence in a group of premenopausal Greek women to evaluate the possible link between AMH-values and the severity of climacteric symptoms during a follow-up of 24 months. This study included 180 women (group A, 96 women of late reproductive stage/early perimenopause; group B, 84 women in late perimenopause). We measured AMH blood levels and assessed climacteric symptoms using the Greene scale. Log-AMH is inversely associated with postmenopausal status. The AMH cut-off of 0.012 ng/mL predicts the postmenopausal status with a sensitivity of 24.2% and specificity of 30.5%. The postmenopausal stage associated with age (OR = 1.320, 95%CI: 1.084–1.320) and AMH (values ≥ vs. <0.012 ng/mL, OR = 0.225, 95%CI: 0.098–0.529, *p*-value < 0.001). Moreover, the severity of vasomotor symptoms (VMS) was only associated inversely with AMH (b-coefficient = −0.272, *p*-value = 0.027). In conclusion, AMH levels measured in the late premenopausal period are inversely associated with the time to ovarian senescence. In contrast, AMH levels measured in the perimenopausal period are inversely associated only with the severity of VMS. Therefore, a cut-off of 0.012 ng/mL predicts menopause with low sensitivity and specificity, making it challenging to use in a clinical setting.

## 1. Introduction

Natural menopause is estimated to occur worldwide at 50.5 years due to primordial follicle pool depletion [1,2]. However, the onset of the menopausal transition appears to vary between different races and ethnicities and is influenced by sociodemographic and lifestyle factors [3]. Moreover, due to the potential social and clinical implications of age at natural menopause (ANM), ANM prediction tools and methods have been a topic of great interest in menopause-related research in recent years [1]. Apart from the general population, prediction of the estimated time of ovarian senescence is an essential tool for cancer survivors [4], as well as in certain disorders of sex development like Turner’s syndrome [5] and women with suspected premature ovarian insufficiency [6]. Thus, estimating the individual ANM and the remaining reproductive years could allow women to make informed family planning decisions [7].

Several tools are being used to identify the time to ovarian senescence. Increasing follicle-stimulating hormone (FSH) and decreasing estradiol levels, as well as the onset of vasomotor symptoms (VMS), namely hot flashes, night sweats, and irregular menstrual cycles, have been long associated with the menopausal transition [8]. However, even though FSH levels accurately represent a woman’s current reproductive state, they have little value in predicting upcoming changes [9,10,11]. The menopausal transition occurs when the primordial follicle pool reaches a critical threshold below which the ovary can no longer produce mature oocytes. At that point, ovarian reserve markers like antral follicle count and anti-Müllerian hormone (AMH) that indirectly reflect the ovarian reserve are suggested to offer an accurate prediction of individual age in natural menopause [12,13]. In addition, the role of the maternal ANM is an essential determinant of the ‘individual’s menopausal age and can offer a more accurate prediction in combination with ovarian reserve markers [14].

Even though AMH appears to be a valuable marker of ovarian reserve, a vast amount of data has demonstrated a recognizable variability in AMH levels. An earlier study showed that the average intraindividual AMH variability was estimated as 20%, the analytical variation as 6.9%, and the biological variation as 19% [15]. Consequently, the variability in AMH levels is likely to result in the reclassification of the individual woman from the high to low AMH group and vice versa, limiting the use of this marker in clinical praxis [15]. Many factors have been associated with this variability, like the prior use of drugs and, more specifically, oral contraceptives, metformin, vitamin D, or gonadotropin-releasing hormone agonists [16]. Other factors that may affect levels of AMH are the phase of the cycle, as AMH levels in the follicular phase were found to be higher than in the luteal phase, with an estimated variability of 11.5% throughout the menstrual cycle [17] as well as the ethnicity or additional factors including the socioeconomic status and environmental parameters [18,19,20,21].

The observed analytical variability in AMH measurements has challenged the technical characteristics of laboratory assays [22]. Until a few years ago, AMH was assessed using manual enzyme-linked immunosorbent assays (ELISAs). However, the ELISA-based assays exhibited considerable between-laboratory variability due to the lack of automation and differences in the completion of specific laboratory procedures [23]. More specifically, the imprecision of the ELISA assays in measuring AMH concentrations was estimated as <6–14%, while there was a considerable difference in the limit of detection, time of incubation (2.5 up to 4.5 h), and measurement range [23]. More recently, the use of automated Elecsys assays started becoming increasingly popular. The Elecsys assays have the benefit of a shorter testing time, estimated as 18 min, and provide reproducible measurements of ovarian reserve. In addition, the automated AMH measurements correlate well with antral follicle count and manual AMH measurements but have less variability [24]. However, the lack of an international standardized protocol to ensure uniform calibration of available AMH assays remains a significant cause of variability between the assessed methods of AMH testing [25].

Considering the possible ethical differences in AMH levels, this study aims to investigate the role of AMH measured using an automated assay in a group of pre-/perimenopausal Greek women, including the most eligible cut-off value of AMH, to detect ovarian senescence. Secondarily, we aimed to evaluate associations between AMH and perimenopausal symptomatology.

## 2. Materials and Methods

This study consisted of 180 consecutively recruited healthy Greek women from the database of consecutive outpatients from the Menopause Clinic, 2nd Department of Obstetrics and Gynaecology, Aretaieion Hospital, National and Kapodistrian University of Athens, Greece. The unit is a tertiary service for peri- and postmenopausal women. All women visiting the clinic are assessed for menopause-related complaints and the possible presence of chronic problems related to aging, like cardiovascular disease and osteoporosis. 

During the first clinic appointment, the personal history is recorded. In addition, we documented information on anthropometric and demographic parameters, lifestyle, physical activity, reproductive history, and other health-related factors (i.e., medical treatment for dyslipidemia, hypertension, or diabetes mellitus). All women are advised to complete the Greene Climacteric Scale (GCS) questionnaire, translated into Greek. 

At the same time, a blood test is done for biochemical and hormonal assessment. The samples are centrifuged and stored in a cold environment (−90 °C) until further assessment. As per the local protocol, women are also offered regular investigations, namely breast mammography, gynecological examination with Papanicolaou smear (as required), and bone density measurement. Following the first consultation, all women receive instructions for a follow-up visit at six or 12 months, depending on their wishes and their needs. 

Data were collected between March and August 2022 by reviewing electronic files for all eligible women. These women were evaluated between 2005–2010 in the menopause clinic of our hospital. All women with a complete data file at baseline and at least one follow-up visit at 12 and/or 24 months were eligible to participate in the study. Time from assessment until the final menstrual period (FMP) was retrospectively evaluated for all women assessed. Women who were still normally menstruating at the time of the blood test were considered as group A (late reproductive stage/early perimenopause) (N = 96). Women experiencing menopausal symptoms and having had their FMP during the last 1–6 months before the study were considered group B (late perimenopause) (N = 84). 

Inclusion criteria were the documented report of regular menstruation or oligomenorrhoea within the 12 months before the baseline assessment in the menopause clinic, as per the patients’ files. Exclusion criteria were: (i) polycystic ovary syndrome, Cushing’s syndrome, or congenital adrenal hyperplasia; (ii) premature ovarian insufficiency diagnosed prior to the start or during the duration of the study (POI); (iii) gynecological malignancy; (iv) inflammatory diseases, (v) oophorectomy or surgical hysterectomy, (vi) adherence or retention concerns, (vii) intake of MHT or selective estrogen receptor modulators, (viii) iron, vitamin B12, folate deficiency, or other types of anemia and electrolyte disturbances. 

Weight was measured, and body mass index (BMI) was calculated as weight (kg)/(height, m)^2^. Waist and hip circumference were measured, and the waist-to-hip ratio was calculated. Arterial pressure was measured non-invasively using an aneroid gauge and semi-automated methods. Hypertension was defined as resting blood pressure > 140/90 mm Hg, measured on at least three occasions or for patients on anti-hypertensive treatment. The menopausal status was defined as a personal history of amenorrhea for ≥12 consecutive months. Women with a report of amenorrhea of shorter intervals were defined as perimenopause. POI was defined as menstrual irregularity in women with follicular stimulating hormone (FSH) levels > 30 IU/L on two occasions, at least 4–6 weeks apart. All participants signed informed consent; the Ethics Committee of the Aretaieion Hospital obtained institutional review board approval.

### 2.1. Greene Climacteric Scale 

The Greene Scale [26] allows for assessing the severity of menopausal symptoms. The scale consists of four main areas, which quantify the intensity of the following symptoms: (a) psychological (items 1–11), (b) physical and psychosomatic (items 12–18), (c) vasomotor (items 19, 20), and (d) sexual (item 21). All 21 items acquire values retrieved from a Likert scale, ranging from 0 to 3 (“not at all”, “occasionally”, “frequently”, or “extremely”), which correspond to a total score out of 63). 

### 2.2. Biochemical and Hormonal Assays 

Total serum cholesterol was measured by enzymatic assay (Abbot, IL, USA) total coefficient of variation of ≤3% sensitivity 5.0 mg/dL). Triglycerides were assessed using the enzymatic glycerol phosphate oxidase methodology (Abbott) (total coefficient of variation of ≤5%, sensitivity 5.0 mg/dL). The ultra-high-density lipoprotein (HDL-C) assay (Abbott, IL, USA) was used to measure HDL-C (total coefficient of variation ≤ 4%, sensitivity of 2.5 mg/dL) due to divergent results detected between various HDL molecules. Other high-density particles (HDL-A, HLD-B) and low-density lipoprotein (LDL) were measured by elimination methodology multiagent direct LDL/HDL, Abbott, IL, USA) (total coefficient of variation less than 4%, sensitivity ≤ 10 mg/dL). Serum glucose was measured by the hexokinase/glucose-6-phosphate dehydrogenase methodology (Abbott) (total coefficient of variation was ≤5%, sensitivity 2.5 mg/dL). We measure serum insulin and sex hormone concentrations [estradiol, FSH, luteinizing hormone (LH)]. All the assays mentioned before were performed on the Architect c8000 system (Abbott Diagnostics, Abbott, IL, USA). AMH levels were analyzed using Elecsys AMH Plus assay (Roche Diagnostics, Meylan, France), a one-step sandwich method based on electrochemiluminescence immunoassay (ECLIA) technology, applied on COBAS e411 analyzer and different COBAS modules (e601, e602 e801). Measuring range 0.07 164 pmol/L (0.01 23 ng/mL) (defined by the limit of detection and the maximum of the master curve). Values below the limit of detection are reported as 164 pmol/L (>23 ng/mL) or up to 328 pmol/L (46 ng/mL) for two-fold diluted samples. The homeostasis model assessment of insulin resistance was calculated using the equation HOMA-IR = fasting glucose (mg/dL) × fasting insulin (mU/L) divided by 405.

### 2.3. Statistical Analysis

All hormone measurements and AMH levels were assessed for normality of the distribution using the Kοlmogorov Smirnov test and visual inspection. However, levels of AMH were not normally distributed. Hence, we proceeded with the assessment using the logarithmically transformed values of AMH. Normally distributed continuous variables were described using mean values and standard deviation (SD), while categorical variables were expressed as frequencies (%).

For the first part of the study, we evaluated the association between values of AMH and time to FMP. For this purpose, a receiver operating characteristic (ROC) curve was used to plot AMH values according to time until FMP. Consequently, we aimed to estimate AMH’s most eligible cut-off value to detect ovarian senescence; Cox regression analysis models were fitted to confirm associations between AMH cut-off values and the time until FMP. Moreover, Kaplan–Meier analysis was used to estimate the time to FMP based on the estimated cut-off for AMH, and the results were compared using a log-rank *p*-value.

For the second part of the study, the mean values of Greene scores (total and subscales) were compared between baseline and follow-up time points (at 12 months and 24 months) using analysis of variance (ANOVA) for paired samples. Correlation analysis was used to estimate possible associations between the severity of vasomotor symptoms and AMH levels, in peri- and premenopausal women, at baseline and 12 and 24 months of follow-up. In addition, multivariable logistic regression analysis was used to assess the possible link between AMH values and the severity of VMS, adjusted for potential risk factors.

Statistical significance was set at *p*-value < 0.05 (two-sided). The Statistical Package for Social Sciences (IBM SPSS) program, version 25.0, was used for statistical analysis. 

## 3. Results

The baseline characteristics of the study population, considered eligible for the current analysis, are presented in Table 1. Group A consisted of 96 women who were assessed (median Age 50 years, range 34 to 55 years) who experienced menopause in 1 up to 174 months after the timing of the blood sampling. Group B consisted of 84 women with a median age of 49 years (range 32–59), median levels of FSH 48.3 mIU/mL (2.5 to 140), median LH 32.8 mIU/mL (1.5 to 99.4), and median estradiol 60.15 pg/mL (5.0 to 334).

**Figure 1:**
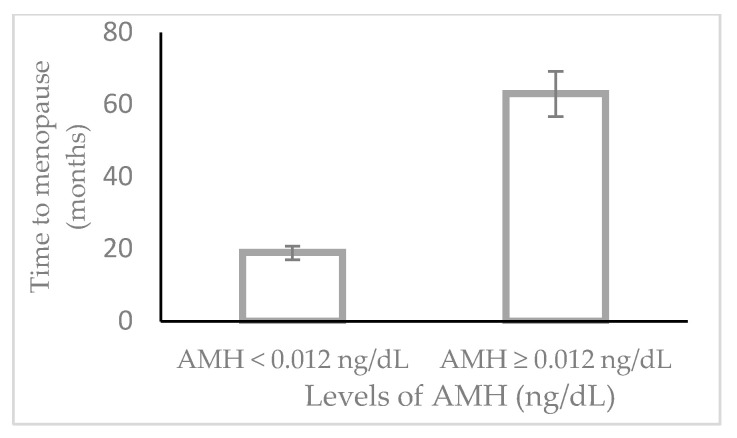
Time to menopause in women with AMH < 0.012 ng/dL or AMH ≥ 0.012 ng/dL. Cox-regression analysis showed that the progress into the menopausal stage associated with age (OR = 1.320, 95%CI: 1.084 to 1.320) and AMH values (values ≥ vs. < 0.012 ng/mL, OR 0.225, 95%CI: 0.098 to 0.529), both cases (*p*-value < 0.001).

Cox regression analysis models were fitted to assess the possible link between values of log-AMH and the diagnosis of menopause, taking into account the time until the FMP. The results are presented in Table 2. Accordingly, values of log-AMH are inversely associated with the postmenopausal status (Model 1, OR 0.110, 95%CI: 0.022 to 0.533). The results remained significant after adjusting for the age of the participants. We applied the ROC curve analysis to estimate the AMH value that better predicts the postmenopausal status. Accordingly, we observed that AMH of 0.012 ng/mL predicted the postmenopausal status with a sensitivity of 24.2% and specificity of 30.5%. Furthermore, the cox-regression analysis showed that the progress into the menopausal stage associated with age (OR 1.320, 95%CI: 1.084 to 1.320) and AMH values (Figure 1 and Figure S1, values ≥ vs. < 0.012 ng/mL, OR 0.225, 95%CI: 0.098 to 0.529), with statistical significance for both cases (*p*-value < 0.001).

Subsequently, we used the Kaplan–Meier Curve to time—to—final menstrual period independently for women with AMH higher vs. lower than the suggested cut-off of 0.012 ng/mL. Accordingly, women with AMH ≥ 0.012 ng/mL vs. women with AMH < 0.012 ng/mL experienced menopause at a longer time-interval (high vs. low AMH), median for time to FMP: 63 ± 22.9 months vs. 19 ± 3.9 months (log-rank *p*-value = 0.001).

Moreover, Table 3 shows the results of the correlation analysis between serum levels of AMH (assessed at baseline) and the severity of climacteric symptoms at baseline and 12 months and 24 months follow-up. We observed that AMH values correlated negatively with the severity of VMS at baseline (r-coefficient = −0.321, *p*-value = 0.014). However, there was no association between baseline levels of AMH and the severity of psychological scores and somatic or sexual scores at baseline, 12 months, or 24 months.

Finally, Table 4 shows the stepwise linear regression analysis results between serum AMH levels and VMS severity at baseline only for the perimenopausal population (group B). The model showed that the severity of VMS was associated inversely with AMH (b-coefficient = −0.272, *p*-value = 0.027) in a model adjusted for age, BMI, HOMA-IR, current smoking, and FSH.

## 4. Discussion

The results of this study explored the possible utilization of AMH values in a sample of Greek pre-and perimenopausal women. First, we confirmed that this marker is reliable in predicting time-to-FMP. A cut-off of 0.012 ng/mL is related to a low-moderate sensitivity and specificity for predicting the time to ovarian senescence. Secondarily, we demonstrated a cross-sectional correlation between baseline AMH values, which were inversely related to the severity of VMS only for perimenopausal group B, but not for premenopausal group A. No association was identified between values of AMH and the severity of any of the remaining climacteric symptoms at baseline or follow-up.

Our results regarding the predictive value of AMH as a marker to assess the time-to-FMP are in accordance with recent literature [11,14,27,28,29,30,31,32,33]. Depmann et al. evaluated the utility of AMH in an extended prospective follow-up study with a mean follow-up period of 14 years [34]. Consolidating their previously published results [10], the authors demonstrated that age and ovarian reserve tests are significantly associated with time to menopause (TTM) and that AMH alone is an independent predictor of TTM. In addition, several population-based studies compared ANM values according to models exhibiting the rate of decline in AMH. Accordingly, the distribution of AMH values has been consistently associated with the time to menopause [35,36,37]. A recent systematic review of 41 studies and 28,858 women evaluated the evidence of using AMH alone or in combination with other markers to predict ovarian senescence [38]. They showed that low AMH was a predictor of menopause equivalent to raised FSH in diagnostic terms, while lower AMH concentrations adjusted for age were associated with an earlier age at menopause. The time to predict ovarian senescence was more accurately estimated by levels of AMH adjusted for age rather than AMH only [38]. A genome-wide meta-analysis, including 3344 premenopausal women retrieved from five cohorts, reported that for the increase in the age of genetically predicted menopause by one year, the age-adjusted AMH was increased by 0.18 SD (95%CI: 0.14 to 0.21) [39].

Despite the consistent evidence that AMH is a more accurate predictor of time-to-FMP than different ovarian reserve tests or mother’s ANM, its clinical use remains conflicting [34,38,40]. In an earlier study of 155 normo-ovulatory women, who were followed up for an average duration of 14 years, AMH had a reduced predictive effect as the age of the women increased [34]. Similarly, as reported by Depmann et al. (2018), in a meta-analysis that included 2596 women, the model assessing time to late menopause (≥55 years of age) demonstrated no added effect of AMH to the model assessing age alone [40]. The same authors reported that the model assessing time to early menopause was improved after incorporating AMH into the model [40]. On the contrary, a recent systematic review reported that the use of the most updated AMH assays had been proven to be useful in the assessment of imminent ovarian senescence. Nevertheless, the prediction of ANM remains imprecise if menopause is not imminent [38].

Identifying a consistent AMH level, which could be used as a cut-off to predict early or premature ovarian senescence, remains conflicting. A Depmann et al. meta-analysis shows that the predictive capacity of models based on age alone is increased by adding AMH levels [40]. Bertone-Johnson et al. showed that the risk of early menopause increased by 14% per 0.10 ng/mL drop in AMH values (95% confidence interval (CI) 1.10–1.18; *p* < 0.001) after evaluating a USA-based population. The risk for early menopause was found to be higher using the specific AMH cut-offs, namely 2.6-times higher using the AMH level of 1.5 ng/mL as a cut-off, 7.5-times higher using the AMH level of 1.0 ng/mL as cut-off and 23-times higher using the AMH level of 0.5 ng/mL as cut-off (all *p* < 0.001) [41]. De Kat et al. evaluated 2434 premenopausal women from the Netherlands, with AMH measurement every 5 years for a follow-up period of 20 years [42]. They described that the rate of AMH decline has a low discriminative ability to estimate the risk of early menopause [42]. Conversely, an analysis of the Tehran Lipid and Glucose Study population showed that the rate of AMH decline is the most effective predictor of early menopause [43]. They demonstrated that a 1-unit increase in the yearly AMH decline rate was associated with a 10.8-times higher risk of early menopause [43]. 

Different AMH assays and the need for a distinct cut-off value constitute pitfalls in the clinical use of AMH. In the SWAN (Study of Women’s Health Across the Nation) study, the sensitivity of a cut-off value of AMH < 10 pg/mL for having the FMP in the next 12 months was 71%, 73%, and 82% in women <48, 48 to <51, and >51 years of age. The positive predictive values for the FMP occurring in the next 12 months were 51%, 63%, and 79% in the three age groups and increased to 78%, 89%, and 97% when the prediction was extended to 36 months [44]. Tehrani et al. found that a cut-off value of 0.39 ng/mL for AMH had the optimal combined sensitivity and specificity for prediction with a positive predictive value of 0.90 (95%CI, 0.81 to 0.96) and negative predictive value of 0.76 (95%CI, 0.65 to 0.86). In a different study, the median time to menopause for 45–48-year-old women with AMH levels lower than 0.20 ng/mL was 5.99 years (95%CI, 4.20 to 6.33) and 9.94 (95%CI, 3.31 to 12.73) for women aged 35–39 years [30].

An additional finding of our study was the inverse cross-sectional association of AMH levels and VMS severity in the premenopausal Greek population, even after adjusting for cardiovascular risk factors. This finding was for the irregular menstruating group B but not the regular menstruating patients. Dhanoya et al. also found that higher levels of AMH were associated with decreased occurrence of hot flushes occurred in the past 2 weeks (OR 0.19; 95%CI 0.05 to 0.69) in a relatively small sample of 108 women aged 40–59 years [45]. Our results also present that baseline values of AMH were not significantly correlated with Greene scores of the psychological, somatic, or sexual subscale, neither in the group of group A nor group B. In a cohort of 2041 premenopausal women aged 42–52 years without any VMS at baseline, the investigators reported an inverse association between quartiles of AMH and the risk for climacteric symptoms during a follow-up period of 4.4 years. More specifically, the multivariable-adjusted hazard ratio (HR) for the development of incident early onset VMS was estimated as 1.02 (95%CI: 0.78 to 1.33) compared to 2.38 (95%CI: 1.84 to 3.08), for women with AMH levels in the top as opposed to the lowest quartile [46]. 

In an attempt to evaluate the link between AMH and the future development of climacteric symptoms, others tried to explore the role of this marker in the population of surgically induced menopause. A small Dutch study evaluated 91 premenopausal women who underwent risk-reducing salpingo-oophorectomy due to their high risk of ovarian cancer [47]. The authors reported that presurgical AMH levels were not related to the change in psychological distress or endocrine and sexual symptoms following surgery, either at 6 weeks or at 7 months [47]. A smaller cohort study investigated the development of menopausal symptoms over time in 170 young women who underwent chemotherapy at least 12 months ago and compared the observations with 135 similar-aged controls and 71 controls of late reproductive age [48]. This study showed that in this group of cancer survivors, ovarian reserve, as reflected by levels of antral follicle count, AMH, and menstrual regularity, could not discriminate the experience of menopausal symptoms during a follow-up of 1.1 up to 11.3 years [48]. On the contrary, a small prospective cohort study evaluated young females with a new cancer diagnosis to assess the role of AMH in predicting symptoms of menopause [49]. Immediately after completing a 3-monthly chemotherapy session, 124 cancer survivors were recruited and compared with 133 similar-aged controls [49]. This study showed that decreased AMH levels after the completion of chemotherapy treatment were related to a 2.2-times higher risk of experiencing vasomotor symptoms, 3-times higher risk for low libido, 2.4-times higher risk for mood swings, 2.7-times higher risk for troubled sleep [49]. 

Our study is the first to explore the possible utilization of AMH for time-to-FMP in the Greek premenopausal and to set a threshold value for that specific population. However, the low specificity and sensitivity do not allow for reliable use in the clinical setting and for making therapeutic decisions based on this marker alone. Recent evidence described evidence of substantial ethnic and racial variability in the reserve of ovarian function, as determined by AMH [18]. For example, the age-related decline in AMH levels has Caucasian compared to African American women [19,20]. Consequently, ethnic-specific AMH reference ranges might be worth considering to improve the accuracy of AMH in predicting ovarian response [18,19,20]. The ethnic-specific variation in levels of AMH appears to be modified by the effect of additional factors like socioeconomic status and nutritional but also environmental parameters [21]. 

In addition, our study has certain limitations, such as the retrospective design, which cannot be avoided given the need for a retrospective diagnosis of menopause 12 months post-FMP.

In summary, we have shown that values of log-AMH are inversely associated with postmenopausal status in Greek women. The association remained significant after adjusting for the age of participants. Women with AMH ≥ 0.012 ng/dL vs. women with AMH < 0.012 ng/dL experienced menopause after a longer time interval. Finally, our results show that AMI values are inversely associated with the severity of VMS but not with the overall severity of climacteric symptoms or the remaining groups of climacteric symptoms.

## Figures and Tables

**Table 1:** Baseline characteristics of the study population.

	Mean ± SD or Frequency (%)	Mean ± SD or Frequency (%)
Anthropometric and demographic parameters	Group A (N = 96)	Group B (N = 84)
Age (years)	48.5 ± 4.5	48.9 ± 4.0
BMI (kg/m^2^)	26.4 ± 4.8	27.1 ± 5.5
Current smoking (%)	26.0% (25/96)	32.3% (27/84)
Current alcohol consumption (%)	50.0% (48/96)	48.4% (40/84)
Climacteric symptoms		
Vasomotor symptoms severity	2.2 ± 1.9	1.9 ±1.9
Hormonal parameters		
AMH (ng/dL)	0.09 ± 0.15	0.01 ± 0.15
FSH (mIU/mL)	25.8 ± 22.2	71.4 ± 26.4
LH (mIU/mL)	23.8 ± 19.0	41.7 ± 15.5
E2 (pg/mL)	123.5 ± 72.3	14.8 ± 12.2
Biochemical parameters		
Total Cholesterol (mg/dL)	217.5 ± 35.0	218.1 ± 35.7
Triglycerides (mg/dL)	81.6 ± 33.9	93.9 ± 44.2
HDL-cholesterol (mg/dL)	59.5 ± 12.0	58.9 ± 13.6
LDL-cholesterol (mg/dL)	136.1 ± 34.1	133.3 ± 31.7
Glucose (mg/dL)	91.8 ± 8.6	95.6 ± 22.6
Insulin (μU/L)	7.2 ± 4.7	7.7 ± 4.7
HOMA-IR	1.7 ± 1.2	1.8 ± 1.0
E2 (pg/mL)	123.5 ± 72.3	14.8 ± 12.2

Note: Group A = late reproductive stage/early perimenopause; Group B = late perimenopause.

**Table 2:** Cox regression analysis to evaluate the time-related change in log-transformed values of anti-Müllerian hormone levels (AMH) until the final menstrual period for women of our sample).

	Chi-Square	Exp (B)	*p*-Value	95%CI
**Model 1** AMH				
	8.454	0.110	**0.007**	0.022–0.553
			
**Model 2** AMH Age (years)				
	22.660	0.069	**0.005**	0.011–0.439
		1.216	**0.001**	1.088–1.360

AMH = anti-Müllerian hormone; CI = confidence interval; Model 1: Cox regression model including AMH levels; Model 2: Model 1 + age (confounder); Bold indicates statistical significance, which was set at the level of *p*-value < 0.05.

**Table 3:** Correlation analysis between serum levels of AMH and climacteric symptoms at baseline, year 1, and year 2 after assessment, using Pearson test.

Greene Scale Scores	Baseline		12 Months		24 Months	
	r-Coefficient	*p*-Value	r-Coefficient	*p*-Value *	r-Coefficient	*p*-Value **
**Vasomotor scores**						
Group A	−0.060	0.754	−0.215	0.312	0.009	0.970
Group B	**−0.321**	**0.014**	0.106	0.487	−0.169	0.363
Total sample	−0.103	0.220	−0.081	0.393	0.028	0.818
**Psychological scores**						
Group A	0.061	0.757	−0.084	0.696	−0.061	0.804
Group B	0.153	0.266	0.165	0.279	0.153	0.420
Total sample	−0.089	0.305	−0.171	0.070	−0.034	0.781
**Somatic scores**						
Group A	0.281	0.139	−0.143	0.504	−0.160	0.553
Group B	0.124	0.367	0.293	0.051	0.118	0.542
Total sample	0.018	0.831	−0.079	0.402	−0.080	0.527
**Sexual scores**						
Group A	0.276	0.139	−0.329	0.117	−0.289	0.231
Group B	0.097	0.473	0.061	0.689	−0.007	0.971
Total sample	0.063	0.459	−0.094	0.320	−0.047	0.695

* Indicates correlation between 12 months of follow-up vs. baseline; Group A = late reproductive stage/early perimenopause; Group B = late perimenopause; ** Indicates correlation between 24 months of follow-up vs. baseline; bold corresponds to statistical significance, set at the *p*-value < 0.05.

**Table 4:** Stepwise linear regression analysis between serum levels of AMH and the severity of vasomotor symptoms at baseline for the perimenopausal population (group B = late perimenopause).

	Model R^2^	b-Coefficient	95%CI	*p*-Value
AMH	7.4%	**−0.272**	**−2.035 to −0.312**	**0.027**
Age (years)		−0.173	−1.384 to 0.234	0.152
BMI (kg/m^2^)		−0.002	−0.473 to 0.984	0.984
HOMA-IR		−0.035	−1.382 to 0.029	0.771
Current smoking		0.141	0.081 to 0.426	0.246
FSH (mIU/mL)		0.054	−0.028 to 0.782	0.671

AMH = anti-Müllerian hormone; BMI = body mass index; HOMA-IR = homeostasis model assessment of insulin resistance; FSH = follicle stimulating hormone; Bold indicates statistical significance, which was set at the level of *p*-value < 0.05.

## Data Availability

The data presented in this study are available on request from the corresponding author. The data are not publicly available due to restrictions by the regulations of the Ethics Committee.

## Supplementary Materials

The following are available online at https://www.mdpi.com/article/10.3390/geriatrics8030057/s1, Figure S1: Flow-chart of data collection.
