# Supplementary material for: Antimüllerian Hormone as a Tool to Predict the Age at Menopause
Source: Geriatrics (Basel). 2023 May 19;8(3):57. doi: 10.3390/geriatrics8030057 (PMC10204528; doi:10.3390/geriatrics8030057)
Supplement: Supplementary file 1 [file geriatrics-08-00057-s001.zip › geriatrics-2168427-supplementary.pdf]

geriatrics MDPI

Supplemental Figure S1. Flow-chart of data collection.

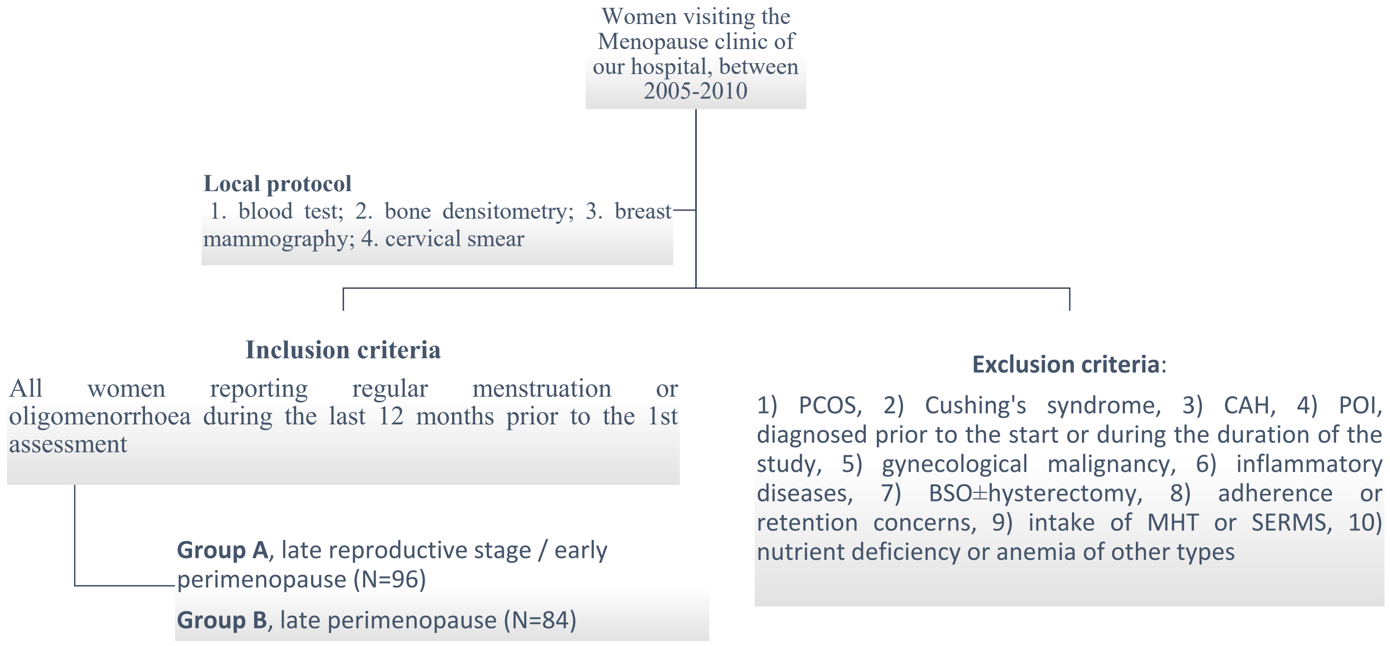

Note: PCOS = polycystic ovary syndrome; CAH = congenital adrenal hyperplasia; POI = premature ovarian insufficiency; BSO = bilateral salpingoophorectomy; MHT = menopause hormone therapy; SERMS = selective estrogen receptor modulators
